# 3,3′-Dimethyl-4,4′-(hexane-1,6-di­yl)bis­[1*H*-1,2,4-triazol-5(4*H*)-one]

**DOI:** 10.1107/S1600536810037311

**Published:** 2010-09-25

**Authors:** Reşat Ustabaş, Ufuk Çoruh, Dilek Ünlüer, Tuncer Hökelek, Emel Ermiş

**Affiliations:** a Department of Middle Education, Educational Faculty, Ondokuz Mayıs University, 55200 Atakum, Samsun, Turkey; b Department of Computer Education and Instructional Technology, Educational Faculty, Ondokuz Mayıs University, 55200 Atakum, Samsun, Turkey; c Department of Chemistry, Faculty of Arts and Sciences, Karadeniz Teknik University, 61080 Trabzon, Turkey; d Department of Physics, Hacettepe University, Beytepe 06800, Ankara, Turkey; e Anadolu University, Faculty of Science, Department of Chemistry, 26470 Yenibaĝlar, Eskişehir, Turkey

## Abstract

The title compound, C_12_H_20_N_6_O_2_, has a centre of symmetry. The mol­ecule consists of two triazole rings joined by an aliphatic –(CH_2_)_6_– chain. The crystal structure is stabilized by inter­molecular N—H⋯O hydrogen bonds and by π–π stacking inter­actions between the triazole rings of inversion-related mol­ecules [centroid–centroid distance = 3.277 (8) Å].

## Related literature

For background information including pharmacological studies, see: Chiu & Huskey (1998 ▶); Clemons *et al.* (2004 ▶); Dalloul & Boyle (2006 ▶); Eliott *et al.* (1986 ▶); Griffin & Mannion (1986 ▶); Santen (2003 ▶); Tanaka (1974 ▶); Zamani *et al.* (2003 ▶). Related structures have been reported by Ustabaş *et al.* (2006 ▶, 2007 ▶, 2009 ▶); Ünver *et al.* (2008 ▶, 2009 ▶); Çoruh *et al.* (2003 ▶).
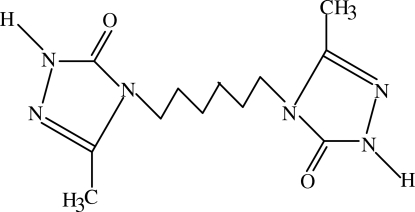


## Experimental

### 

#### Crystal data

C_12_H_20_N_6_O_2_
*M* *_r_* = 280.34
Triclinic, 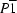

*a* = 6.3641 (2) Å
*b* = 7.3034 (2) Å
*c* = 7.7774 (2) Å
α = 93.299 (2)°
β = 109.578 (2)°
γ = 94.707 (2)°
*V* = 338.05 (2) Å^3^
*Z* = 1
Mo *K*α radiation
μ = 0.10 mm^−1^
*T* = 101 K
0.40 × 0.16 × 0.12 mm

#### Data collection

Bruker Kappa APEXII CCD area-detector diffractometer
Absorption correction: multi-scan (*SADABS*; Bruker, 2007 ▶) *T*_min_ = 0.962, *T*_max_ = 0.988
6074 measured reflections
1673 independent reflections
1309 reflections with *I* > 2σ(*I*)
*R*_int_ = 0.033

#### Refinement

*R*[*F* ^2^ > 2σ(*F* ^2^)] = 0.041
*wR*(*F* ^2^) = 0.112
*S* = 1.03
1673 reflections
131 parameters
All H-atom parameters refined
Δρ_max_ = 0.32 e Å^−3^
Δρ_min_ = −0.28 e Å^−3^

### 

Data collection: *APEX2* (Bruker, 2007 ▶); cell refinement: *SAINT* (Bruker, 2007 ▶); data reduction: *SAINT*; program(s) used to solve structure: *SHELXS97* (Sheldrick, 2008 ▶); program(s) used to refine structure: *SHELXL97* (Sheldrick, 2008 ▶); molecular graphics: *ORTEP-3 for Windows* (Farrugia, 1997 ▶); software used to prepare material for publication: *WinGX* (Farrugia, 1999 ▶).

## Supplementary Material

Crystal structure: contains datablocks global, I. DOI: 10.1107/S1600536810037311/pk2267sup1.cif

Structure factors: contains datablocks I. DOI: 10.1107/S1600536810037311/pk2267Isup2.hkl

Additional supplementary materials:  crystallographic information; 3D view; checkCIF report

## Figures and Tables

**Table 1:** Hydrogen-bond geometry (Å, °)

*D*—H⋯*A*	*D*—H	H⋯*A*	*D*⋯*A*	*D*—H⋯*A*
N3—H3⋯O1^i^	0.90 (2)	1.89 (2)	2.7707 (15)	167 (2)

Symmetry code: (i) 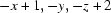.

**Fig. 1.**
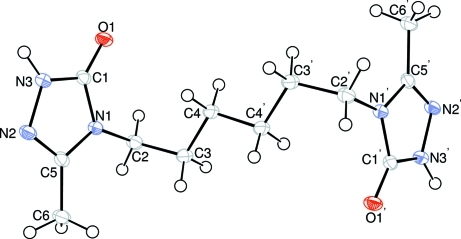
An ellipsoid plot of the title compound, with the atom numbering scheme. Atoms with primed labels are related via an inversion center (1-x, 1-y, 1-z). Displacement ellipsoids are drawn at the 50% probability level.

**Fig. 2.**
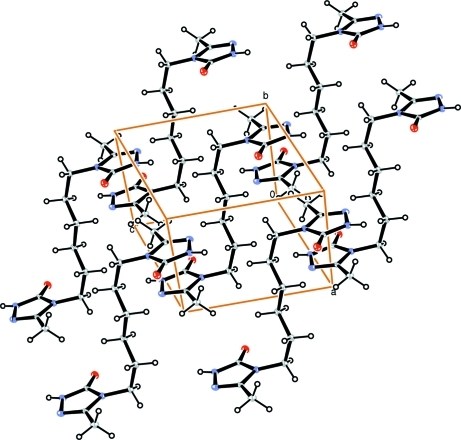
A packing diagram, viewed along b.

## supplementary crystallographic information

### Comment

The 1,2,4-triazole compounds possess important pharmacological activities that include antifungal and antiviral properties. Examples of compounds bearing the 1,2,4-triazole group are fluconazole, the powerful azole antifungal agent as well as the potent antiviral N– nucleoside ribavirin (Ünver *et al.*, 2008; Ünver *et al.*, 2009).  Furthermore, various 1,2,4-triazole derivatives have been reported as fungicidal (Zamani *et al.*, 2003), insecticidal (Tanaka, 1974), antimicrobial (Griffin & Mannion, 1986), and some showed antitumor activity as well as having anticonvulsant (Dalloul & Boyle, 2006), antidepressant (Chiu & Huskey, 1998) and plant growth regulator anticoagulant activity (Eliott *et al.*, 1986). It was reported that compounds having triazole moieties, such as Vorozole, Anastrozole and Letrozole appear to be very effective aromatase inhibitors and can be useful for preventing breast cancer (Santen, 2003; Clemons *et al.*, 2004).

The molecular structure of the compound is shown in Fig.1.  The molecule consists of two triazole rings, joined by an aliphatic —(CH_2_)_6_— chain connected to nitrogen atoms of the rings.  The molecule has an inversion center in the middle of the chain, that connects the triazole rings.  The length of the N═C [N2═C5= 1.3031 (17) Å] bond in the triazole ring is close to the those similar structures in the literature [1.296 (3)Å in C_14_H_16_N_6_O_2_S (Ustabaş *et al.*, 2007); 1.288 (2)Å in C_16_H_28_N_6_O_2_ (Çoruh *et al.*, 2003)]. The bond length of O═C [O1═C1= 1.2421 (16) Å] is in conformity with the values mentioned before[1.218 (3)Å in C_16_H_20_N_6_O_2_S (Ustabaş *et al.*, 2006); 1.220 (2)Å in C_24_H_20_N_4_O_2_S (Ustabaş *et al.*, 2009)]. The triazole ring is very close to planarity, with a maximum deviation from the least-squares plane of -0.014 (13)Å for atom C1.

In the crystal structure of the compound, there is a strong intermolecular N3—H3···O1 hydrogen-bonding interaction (Table 1). The compound also exhibits π-π stacking interactions between triazole rings (*Cg*1···*Cg*1= 3.277 (8) Å; symmetry code: –*X*, 2-Y, –*Z*).

### Experimental

The synthesis of 4,4'-(hexane-1,6-diyl)bis (5-ethyl-2*H*-1,2,4-triazol-3(4*H*)-one) to a solution of ethyl 2 (1-ethoxyethylidene)hydrazinecarboxylate (0.02 mol) in 50 ml water hexane-1,6-diamine (0.01 mol) was added. Having refluxed this mixture for 4 h the precipitate formed was filtered off. The solid product was washed with water and crystallized from ethanol/water (1/3)(yield 73.25%) to afford the desired compound.

### Refinement

All H atoms were located in a difference synthesis and refined [N—H = 0.902 (19) Å; ethylene C—H = 0.945 (18) Å-1.017 (18) Å; and methylene C—H= 0.952 Å-1.00 (2) Å].

### Crystal data

**Table:**

C_12_H_20_N_6_O_2_	*Z* = 1
*M**_r_* = 280.34	*F*(000) = 150
Triclinic, *P*1	*D*_x_ = 1.377 Mg m^−^^3^
Hall symbol: -P 1	Mo *K*α radiation, λ = 0.71073 Å
*a* = 6.3641 (2) Å	Cell parameters from 1309 reflections
*b* = 7.3034 (2) Å	θ = 2.8–28.3°
*c* = 7.7774 (2) Å	µ = 0.10 mm^−^^1^
α = 93.299 (2)°	*T* = 101 K
β = 109.578 (2)°	Rod-shaped, colorless
γ = 94.707 (2)°	0.40 × 0.16 × 0.12 mm
*V* = 338.05 (2) Å^3^	

### Data collection

**Table:**

Bruker Kappa APEXII CCD area-detector diffractometer	1673 independent reflections
Radiation source: fine-focus sealed tube	1309 reflections with *I* > 2σ(*I*)
graphite	*R*_int_ = 0.033
φ and ω scans	θ_max_ = 28.3°, θ_min_ = 2.8°
Absorption correction: multi-scan (*SADABS*; Bruker, 2007)	*h* = −8→8
*T*_min_ = 0.962, *T*_max_ = 0.988	*k* = −9→9
6074 measured reflections	*l* = −10→10

### Refinement

**Table:**

Refinement on *F*^2^	Primary atom site location: structure-invariant direct methods
Least-squares matrix: full	Secondary atom site location: difference Fourier map
*R*[*F*^2^ > 2σ(*F*^2^)] = 0.041	Hydrogen site location: inferred from neighbouring sites
*wR*(*F*^2^) = 0.112	All H-atom parameters refined
*S* = 1.03	*w* = 1/[σ^2^(*F*_o_^2^) + (0.062*P*)^2^ + 0.0594*P*] where *P* = (*F*_o_^2^ + 2*F*_c_^2^)/3
1673 reflections	(Δ/σ)_max_ < 0.001
131 parameters	Δρ_max_ = 0.32 e Å^−^^3^
0 restraints	Δρ_min_ = −0.28 e Å^−^^3^

### Special details

**Table:**

Geometry. All e.s.d.'s (except the e.s.d. in the dihedral angle between two l.s. planes) are estimated using the full covariance matrix. The cell e.s.d.'s are taken into account individually in the estimation of e.s.d.'s in distances, angles and torsion angles; correlations between e.s.d.'s in cell parameters are only used when they are defined by crystal symmetry. An approximate (isotropic) treatment of cell e.s.d.'s is used for estimating e.s.d.'s involving l.s. planes.
Refinement. Refinement of *F*^2^ against ALL reflections. The weighted *R*-factor *wR* and goodness of fit *S* are based on *F*^2^, conventional *R*-factors *R* are based on *F*, with *F* set to zero for negative *F*^2^. The threshold expression of *F*^2^ > σ(*F*^2^) is used only for calculating *R*-factors(gt) *etc*. and is not relevant to the choice of reflections for refinement. *R*-factors based on *F*^2^ are statistically about twice as large as those based on *F*, and *R*- factors based on ALL data will be even larger.

### Fractional atomic coordinates and isotropic or equivalent isotropic displacement parameters (Å^2^)

**Table:**

	*x*	*y*	*z*	*U*_iso_*/*U*_eq_	
O1	0.49741 (15)	0.02539 (13)	0.76514 (13)	0.0199 (3)	
N1	0.84928 (18)	0.18555 (15)	0.81036 (15)	0.0159 (3)	
N2	0.98018 (18)	0.22436 (15)	1.11442 (15)	0.0183 (3)	
N3	0.76344 (18)	0.13460 (15)	1.04956 (15)	0.0173 (3)	
C1	0.6815 (2)	0.10507 (17)	0.86549 (18)	0.0162 (3)	
C2	0.8354 (2)	0.1878 (2)	0.61861 (18)	0.0189 (3)	
C3	0.7642 (2)	0.36813 (19)	0.54057 (19)	0.0202 (3)	
C4	0.5280 (2)	0.40487 (19)	0.53108 (19)	0.0193 (3)	
C5	1.0248 (2)	0.25341 (17)	0.96590 (18)	0.0166 (3)	
C6	1.2408 (2)	0.3456 (2)	0.9639 (2)	0.0207 (3)	
H21	0.981 (3)	0.163 (2)	0.609 (2)	0.019 (4)*	
H61	1.220 (3)	0.461 (3)	0.914 (2)	0.030 (4)*	
H32	0.772 (3)	0.361 (2)	0.415 (2)	0.025 (4)*	
H41	0.512 (3)	0.397 (2)	0.655 (2)	0.018 (4)*	
H31	0.876 (3)	0.476 (2)	0.614 (2)	0.028 (4)*	
H42	0.414 (3)	0.309 (2)	0.446 (2)	0.022 (4)*	
H22	0.732 (3)	0.087 (2)	0.551 (2)	0.025 (4)*	
H3	0.696 (3)	0.089 (2)	1.125 (3)	0.036 (5)*	
H62	1.310 (3)	0.273 (3)	0.887 (3)	0.035 (5)*	
H63	1.348 (3)	0.368 (3)	1.093 (3)	0.041 (5)*	

### Atomic displacement parameters (Å^2^)

**Table:**

	*U*^11^	*U*^22^	*U*^33^	*U*^12^	*U*^13^	*U*^23^
O1	0.0140 (5)	0.0240 (5)	0.0184 (5)	−0.0042 (4)	0.0030 (4)	0.0004 (4)
N1	0.0126 (6)	0.0162 (6)	0.0181 (6)	0.0005 (4)	0.0043 (5)	0.0024 (4)
N2	0.0123 (6)	0.0186 (6)	0.0213 (6)	−0.0005 (4)	0.0028 (5)	0.0012 (4)
N3	0.0127 (6)	0.0192 (6)	0.0183 (6)	−0.0006 (4)	0.0035 (5)	0.0026 (4)
C1	0.0134 (6)	0.0150 (6)	0.0197 (7)	0.0028 (5)	0.0047 (5)	0.0025 (5)
C2	0.0156 (7)	0.0230 (7)	0.0169 (7)	−0.0009 (6)	0.0049 (5)	−0.0001 (5)
C3	0.0163 (7)	0.0237 (7)	0.0202 (7)	−0.0017 (6)	0.0065 (6)	0.0039 (6)
C4	0.0160 (7)	0.0213 (7)	0.0184 (7)	−0.0029 (5)	0.0039 (6)	0.0035 (5)
C5	0.0130 (6)	0.0153 (6)	0.0198 (7)	0.0033 (5)	0.0028 (5)	0.0018 (5)
C6	0.0131 (7)	0.0208 (7)	0.0261 (8)	−0.0005 (5)	0.0045 (6)	0.0021 (6)

### Geometric parameters (Å, °)

**Table:**

O1—C1	1.2421 (16)	C3—C4	1.5271 (19)
N1—C5	1.3751 (17)	C3—H32	0.991 (16)
N1—C1	1.3794 (16)	C3—H31	1.017 (18)
N1—C2	1.4653 (16)	C4—C4^i^	1.528 (3)
N2—C5	1.3031 (17)	C4—H41	1.007 (15)
N2—N3	1.3907 (15)	C4—H42	1.000 (17)
N3—C1	1.3467 (18)	C5—C6	1.4856 (19)
N3—H3	0.902 (19)	C6—H61	0.952 (18)
C2—C3	1.521 (2)	C6—H62	1.006 (18)
C2—H21	0.985 (15)	C6—H63	1.00 (2)
C2—H22	0.945 (18)		
			
C5—N1—C1	107.39 (11)	C2—C3—H31	110.3 (10)
C5—N1—C2	128.60 (11)	C4—C3—H31	109.3 (9)
C1—N1—C2	123.98 (11)	H32—C3—H31	107.2 (13)
C5—N2—N3	103.79 (11)	C3—C4—C4^i^	112.27 (14)
C1—N3—N2	112.63 (11)	C3—C4—H41	110.2 (9)
C1—N3—H3	124.9 (12)	C4^i^—C4—H41	108.2 (8)
N2—N3—H3	122.0 (12)	C3—C4—H42	110.5 (9)
O1—C1—N3	128.98 (12)	C4^i^—C4—H42	109.0 (9)
O1—C1—N1	126.86 (12)	H41—C4—H42	106.5 (13)
N3—C1—N1	104.16 (11)	N2—C5—N1	111.99 (11)
N1—C2—C3	112.31 (11)	N2—C5—C6	124.26 (13)
N1—C2—H21	108.9 (9)	N1—C5—C6	123.74 (12)
C3—C2—H21	111.3 (9)	C5—C6—H61	110.6 (10)
N1—C2—H22	107.6 (10)	C5—C6—H62	113.5 (10)
C3—C2—H22	110.7 (10)	H61—C6—H62	105.9 (14)
H21—C2—H22	105.8 (13)	C5—C6—H63	108.9 (11)
C2—C3—C4	114.14 (11)	H61—C6—H63	108.3 (15)
C2—C3—H32	106.5 (9)	H62—C6—H63	109.6 (14)
C4—C3—H32	109.1 (9)		
			
C5—N2—N3—C1	1.87 (14)	N1—C2—C3—C4	−64.04 (15)
N2—N3—C1—O1	178.11 (12)	C2—C3—C4—C4^i^	174.64 (14)
N2—N3—C1—N1	−2.33 (14)	N3—N2—C5—N1	−0.58 (14)
C5—N1—C1—O1	−178.57 (12)	N3—N2—C5—C6	−179.47 (12)
C2—N1—C1—O1	−0.5 (2)	C1—N1—C5—N2	−0.81 (15)
C5—N1—C1—N3	1.86 (13)	C2—N1—C5—N2	−178.77 (12)
C2—N1—C1—N3	179.93 (11)	C1—N1—C5—C6	178.09 (12)
C5—N1—C2—C3	−84.62 (16)	C2—N1—C5—C6	0.1 (2)
C1—N1—C2—C3	97.73 (14)		

Symmetry codes: (i) −*x*+1, −*y*+1, −*z*+1.

### Hydrogen-bond geometry (Å, °)

**Table:**

*D*—H···*A*	*D*—H	H···*A*	*D*···*A*	*D*—H···*A*
N3—H3···O1^ii^	0.90 (2)	1.89 (2)	2.7707 (15)	167 (2)

Symmetry codes: (ii) −*x*+1, −*y*, −*z*+2.
